# From oral dysbiosis to digital public health pathways: translating Indonesian HIV oral microbiome evidence into integrated care

**DOI:** 10.1080/20002297.2026.2746874

**Published:** 2026-10-13

**Authors:** Deby Febriyan Eprilianto, Yuyun Eka Kartika Sari

**Affiliations:** a Department of Public Administration, Universitas Negeri Surabaya, Surabaya, Indonesia

To the Editor,

The article by Anshory et al. is a timely contribution to oral microbiology and HIV care in Indonesia. By analysing oral rinse samples from 245 adults using 16S rRNA gene sequencing, the authors show that HIV infection is associated with a distinct oral dysbiosis, that ART-naive participants have enrichment of pro-inflammatory genera, and that ART-treated participants partially shift toward HIV-negative oral microbiome profiles [1]. These findings are biologically important. They also raise a translational question that extends beyond laboratory interpretation: how can oral microbiome evidence be converted into integrated, equitable, and digitally enabled public health services for people living with HIV?

This letter proposes that the findings should be read not only as a basis for personalized oral health management, but also as a prompt to design a public health pathway for microbiome-informed HIV care. Three implementation considerations may strengthen the clinical and policy value of this emerging evidence. First, oral microbiome risk should be embedded into HIV service integration. The partial movement of ART-treated participants toward HIV-negative profiles suggests that ART has relevance for oral ecological recovery. At the same time, the persistence of microbial differences indicates that ART alone may not fully remove oral health vulnerability. In Indonesia, this point is particularly salient. A recent WHO-supported HIV Joint Programme Review noted continuing gaps in coordination, service integration, and sustainability in the national HIV response [2]. Within this context, oral health assessment should not remain an optional specialist add-on. Simple oral screening, periodontal risk assessment, oral hygiene counselling, and referral triggers could be incorporated into HIV clinics, primary health centres, and referral hospitals, while microbiome sequencing is reserved for sentinel research and high-risk clinical settings. Such an approach would also align with the WHO oral health country profile for Indonesia, which frames oral health as an urgent public health and universal health coverage concern [3].

Second, digital health governance should be considered part of microbiome translation. Indonesia is currently developing SATUSEHAT as a standardized and integrated national health data ecosystem [4]. This infrastructure creates an opportunity to connect biological insight with service delivery, provided that data use is ethical, proportionate, and clinically meaningful. Future studies could therefore combine microbial measures with implementation variables such as ART status and duration, oral symptoms, periodontal indicators, smoking, diabetes, referral history, health facility type, and geographic access to dental care. These variables do not replace sequencing; they explain how microbial vulnerability becomes service vulnerability. A tiered model may be feasible: routine oral health indicators captured in electronic medical records; clinician-facing referral prompts for high-risk patients; district-level dashboards for service planning; and sentinel oral microbiome surveillance in university or referral laboratories. This would shift the translational frame from individual precision medicine alone toward precision public health.

Third, privacy, stigma reduction, and community trust must be treated as microbiome implementation requirements. HIV-related data are highly sensitive, and microbiome data may become sensitive when linked to immune status, comorbidities, or behavioural risk factors. Digitalisation can improve continuity of care, but it can also amplify discrimination if implemented as broad data collection without consent, data minimisation, role-based access, and transparent governance. For this reason, microbiome-informed oral health programmes for people living with HIV in Indonesia should be co-designed with patient communities, dental professionals, HIV clinicians, public health agencies, and local governments. Community empowerment is not peripheral to microbiological translation; it determines whether patients accept oral examination, attend referrals, and trust data linkage across facilities.

The broader oral microbiome literature increasingly recognises that oral microbial communities are spatially diverse, ecologically dynamic, and relevant to both oral and systemic health [5]. The Indonesian evidence provided by Anshory et al. therefore offers an important opening for locally grounded translational research. Longitudinal studies could clarify whether oral microbial shifts are sustained after ART initiation and whether targeted oral health interventions modify microbial trajectories. Implementation studies could test whether digital reminders, integrated appointment systems, dental referral modules, or community-based oral health education improve screening uptake and follow-up among people living with HIV. In settings where routine sequencing is not yet affordable, combined indicators of oral symptoms, periodontal signs, ART status, and comorbidities can guide prioritisation, while reference laboratories support confirmatory microbiome analyses.

In conclusion, Anshory et al. provide valuable Indonesian evidence that HIV and ART status are associated with oral microbiome composition. The next translational step is to embed this biological insight into integrated, privacy-protecting, and digitally enabled public health pathways. Doing so would strengthen the connection between oral microbiology, HIV service integration, and Indonesia's digital health transformation, while helping to convert microbiome findings into equitable oral health interventions for people living with HIV.

## Data Availability

Data sharing is not applicable to this article because no new data were created or analysed.
